# Characterization of occupational exposure to air pollutants during asphalt milling and paving

**DOI:** 10.1093/annweh/wxaf078

**Published:** 2025-11-20

**Authors:** Maria Hedmer, Karin Lovén, Johannes Rex, Carina A Nilsson, Merve Polat, Jakob K Nøjgaard, Joakim Pagels, Bo Strandberg, Lina Hagvall

**Affiliations:** Department of Occupational and Environmental Medicine, Skåne University Hospital, Lund SE-22381, Sweden; Division of Occupational and Environmental Medicine, Department of Laboratory Medicine, Lund University, Lund SE-22100, Sweden; Department of Occupational and Environmental Medicine, Skåne University Hospital, Lund SE-22381, Sweden; Division of Occupational and Environmental Medicine, Department of Laboratory Medicine, Lund University, Lund SE-22100, Sweden; Ergonomics and Aerosol Technology, LTH, Lund University, Lund SE-22100, Sweden; Department of Occupational and Environmental Medicine, Skåne University Hospital, Lund SE-22381, Sweden; Division of Occupational and Environmental Medicine, Department of Laboratory Medicine, Lund University, Lund SE-22100, Sweden; National Research Centre for the Working Environment, Copenhagen DK-2100, Denmark; Department of Chemistry, University of Copenhagen, Copenhagen DK-2100, Denmark; National Research Centre for the Working Environment, Copenhagen DK-2100, Denmark; Department of Chemistry, University of Copenhagen, Copenhagen DK-2100, Denmark; Ergonomics and Aerosol Technology, LTH, Lund University, Lund SE-22100, Sweden; Department of Occupational and Environmental Medicine, Skåne University Hospital, Lund SE-22381, Sweden; Division of Occupational and Environmental Medicine, Department of Laboratory Medicine, Lund University, Lund SE-22100, Sweden; Department of Occupational and Environmental Medicine, Skåne University Hospital, Lund SE-22381, Sweden; Division of Occupational and Environmental Medicine, Department of Laboratory Medicine, Lund University, Lund SE-22100, Sweden

**Keywords:** biological monitoring, dermal exposure, direct-reading monitors, LDSA, NO_2_, PAHs, particle number, ultrafine particles

## Abstract

**Objectives:**

Asphalt is frequently used as road pavement and consists of bitumen as a binder, and fillers. Bitumen consists of a complex mixture of hydrocarbons, where a minor component is polycyclic aromatic hydrocarbons (PAHs). Many PAHs are classified as carcinogenic to humans. Bitumen fumes from road paving have been classified as possibly carcinogenic. Paving and milling are open processes generating asphalt fumes, mechanically generated dust particulate matter and diesel exhaust, which the asphalt workers are exposed to. Ultrafine particles (UFPs) are present in both asphalt fumes and diesel exhaust. The aim was to characterize occupational exposure of milling and road paving with a novel multi-metric approach by using real-time monitors and offline methods. Additional aims were to monitor asphalt workers' skin contamination of PAHs by skin wiping, and to biologically monitor their systemic exposure to PAH in urine.

**Methods:**

Personal exposure measurements of lung deposited surface area (LDSA), particle number concentration (PNC), particulate mass (PM_0.3_), average particle size, organic carbon (OC), elemental carbon (EC), equivalent black carbon, 16 US Environmental Protection Agency (EPA) PAHs, and nitrogen dioxide (NO_2_) were performed on millers and pavers in a field study. Skin wipe samples (palm) and urine samples were collected before and after workshifts and were analysed for PAH and PAH metabolites, respectively. Repeated self-administered samplings of 16 US EPA PAHs and NO_2_ were performed twice by the millers and pavers.

**Results:**

The pavers had the highest average exposure to all exposure metrics, except for OC and NO_2_. Their geometric mean (GM) exposures to PNC and LDSA were 31,000/cm^3^ and 80 µm^2^/cm^3^, respectively. The GM exposure to 16 US EPA PAHs, OC, EC, and NO_2_ were 0.29, 21, 0.75, and 31 µg/m^3^, respectively. The millers’ GM exposures to PNC and LDSA were 29,000/cm^3^ and 67 µm^2^/cm^3^, respectively. Their GM exposure to 16 US EPA PAHs, OC, EC, and NO_2_ were 0.053, 40, 0.40, and 83 µg/m^3^, respectively. The self-administrated sampling of 16 US EPA PAH and NO_2_ showed that the exposures were in the same range as in the field study, increasing the validity of the results. Pavers showed significantly higher levels of PAH on the palm after the workshift compared with millers. Millers showed higher levels of benzo[a]pyrene on their palm after the workshift compared with pavers. The urinary levels of PAH metabolites were significantly increased in pavers after the workshift.

**Conclusions:**

This study showed that millers and pavers were exposed to airborne 16 US EPA PAHs, UFPs, OC, and diesel exhaust. With a study design that involved repeated exposure measurements for each participant, more accurate exposure characterization and assessment of PAHs and NO_2_ were obtained. By using portable aerosol monitors, valuable exposure data for novel metrics, including UFPs, could be obtained. Operators of, eg, rollers and milling machines were exposed to multiple peak exposures during the workshift. Millers were exposed to somewhat elevated levels of the carcinogenic particulate PAHs. As biomonitoring generally is measuring metabolites of gaseous and intermediate molecular mass PAHs, particulate PAH exposure could not be detected. Air and skin exposure measurements were vital in order to detect this exposure. Recommendations for reducing occupational exposure are proposed.

What's Important About This Paper? This study demonstrates that portable direct-reading monitors can be used to measure novel exposure metrics, like lung deposited surface area and particle number concentration of ultrafine particles in asphalt workers' breathing zones. This study found that both air and skin exposure measurements of polycyclic aromatic hydrocarbons (PAHs) were necessary in order to detect exposure to particulate PAHs, which are carcinogenic compounds, as biomonitoring generally is performed by measurements of metabolites of gaseous and intermediate PAHs.

## Introduction

Asphalt is a material that is frequently used as a road pavement layer. The asphalt mixture consists of bitumen as a binder and gravel or crushed rock, and sand as an inorganic filler (Nilsson et al. 2018; Germin-Aizac et al. 2023). Bitumen is a viscous liquid that consists of a complex mixture of high-molecular-weight hydrocarbons and is produced from crude oil by refining. Bitumen fumes released from asphalt work and paving have been classified as Group 2B (IARC 2011). In bitumen, aliphatic hydrocarbons (C10 to C40) are the major compounds, but other pollutants (eg aldehydes and alcohols) are also present (Germin-Aizac et al. 2023). One minor component group in bitumen is polycyclic aromatic hydrocarbons (PAHs). The PAH content can vary due to its origin of the crude oil, the refinery process, and possible additives (Cavallari et al. 2012; Nilsson et al. 2018). PAHs are a group of compounds that are characterized by the presence of 2 or more fused benzene rings. PAHs occur naturally, but they are also formed by incomplete combustion of organic material or during various industrial processes (Strandberg et al. 2018a). In 1970, the US Environmental Protection Agency (US EPA) identified 16 US EPA PAH compounds as priority pollutants (Zelinkova and Wenzl 2015). Among PAHs in bitumen, benzo[a]pyrene has been classified as carcinogenic to humans (Group 1) by the International Agency for Research on Cancer, while other PAHs are classified as probably carcinogenic (Group 2A; dibenz[a,h]anthracene) or possibly carcinogenic (Group 2B; naphthalene, benzo[a]anthracene, and chrysene) (IARC 2006, 2010, 2014, 2016). The carcinogenicity of PAHs increases with increasing molecular weight, ie the PAHs with high molecular mass (H-PAH) are the most carcinogenic.

According to literature, asphalt workers are mainly exposed to asphalt fumes, diesel exhaust, and road dust (Elihn et al. 2008; Freund et al. 2012). Asphalt milling and asphalt paving of roads are open processes mainly performed outdoors. Both processes generate fumes of bitumen, PAHs, and particulate matter (PM), which the workers are exposed to (Germin-Aizac et al. 2023). It has previously been shown that ultrafine particles (UFPs) were emitted from paving, eg the average diameter of airborne particles at a paving site was 70 nm, while high total dust mass concentrations were emitted from milling (Elihn et al. 2008).

Occupational exposure of asphalt workers has previously been studied using the following airborne exposure metrics to measure exposure, PM (total, inhalable, thoracic, and respirable dust fractions), selected PAHs, organic carbon (OC), elemental carbon (EC), total carbon (TC), selected amines, and oil mist and vapour (Burstyn et al. 2002; Heikkilä et al. 2002; Cirla et al. 2007; Elihn et al. 2008; Cavallari et al. 2012; Osborn et al. 2013; Nilsson et al. 2018; Weiss et al. 2018; Olsen et al. 2021).

In recent years, pocket-sized direct-reading monitors have been used to characterize different occupational exposures; for example, aethalometers have been used to measure diesel exhaust particles (Yu et al. 2015; Gren et al. 2022), engineered carbon nanoparticles (Lovén et al. 2021; Hedmer et al. 2022), and fire smoke particles (Lovén et al. 2024). Furthermore, particle number concentrations (PNCs) have been measured with personal monitors to characterize exposure of diesel exhaust (Hedmer et al. 2017), engineered nanoparticles (Isaxon et al. 2020), metal dust (Lovén et al. 2023), and fire smoke (Lovén et al. 2024). Only a few studies have used portable monitors to characterize lung-deposited surface area (LDSA) of UFPs in the alveolar region for different occupational exposures, such as chimney sweeps, downstream industrial handling of nanomaterials, waste recycling, and training of firefighters (Lovén et al. 2021, 2023, 2024; Moazami et al. 2024). LDSA is an important metric for adverse health effects of particle exposures as it combines the lung deposition and surface area of particles (Liu et al. 2023). In the studied worksites, there was traffic close to the area that was milled or paved, and work machines were commonly diesel-powered. For that reason, occupational exposure to diesel exhaust has been measured as nitrogen dioxide (NO_2_), EC, and equivalent black carbon (eBC), an exposure proxy for EC.

Biomonitoring of PAH metabolites, mainly 1-hydroxypyrene (1-OH-Pyr), has been used extensively to investigate PAH uptake from all exposure routes (inhalation, skin, and ingestion) among occupationally exposed workers such as firefighters (Fent et al. 2014, 2020; Wingfors et al. 2018; Lovén et al. 2024), diesel exposed workers (Gren et al. 2022; Casjens et al. 2023), and asphalt pavers (McClean et al. 2004; Cavallo et al. 2006; Sobus et al. 2009; Fostinelli et al. 2018; Germin-Aizac et al. 2023). However, other PAH metabolites have also been quantified in urine of pavers, eg, monohydroxylated metabolites of naphthalene and phenantrene (Sobus et al. 2009; Germin-Aizac et al. 2023).

Skin exposure has been shown to be a major route of PAH exposure (Keir et al. 2017, 2020; Stec et al. 2018 ; Wingfors et al. 2018). However, in a recent review study, it was stated that the primary routes of occupational exposure to PAHs are inhalation and, to a lesser extent, dermal contact (Clauzel et al. 2024).

### Aim

The aim of this study was to characterize occupational exposure to PM for asphalt millers and asphalt pavers by air monitoring of OC, EC, eBC, as well as LDSA, PNC, particle size, and particulate mass (PM_0.3_) of UFPs. Also, PAHs (both particle-bound and gaseous) and NO_2_ were measured. An additional aim was to monitor asphalt workers' skin contamination of PAHs by skin wiping of the palm of the dominant hand and finally to biologically monitor the systemic exposure to PAH by measuring urinary metabolites of PAHs before and after work.

## Materials and methods

### Workplace

The company included in the study was a larger Swedish paving company performing asphalt milling and asphalt paving of roads. The exposure measurements were performed at 3 locations with milling of roads, 4 locations with asphalt paving of roads, and 1 location where both milling and paving were conducted, see Tables 1 and 2. Information about the teams, vehicles, worksite, and asphalt type at the exposure measurements is also presented in the tables. The meteorological conditions during the field measurements can be seen in Table S1. The field measurements were performed during 8 workdays from the autumn of 2022 to the summer of 2023.

**Table 1.** Description of the performed milling work during the field measurements.

Measurement day	Teams	Vehicles	Worksites
A	Each team (day A-C) consisted of a milling machine operator and an operator of the sweeper, both participated in the study.	Milling machines (Wirtgen W120Ri; W100Ri)^a^ without vehicle cabins Sweepers (1 swing loader Mecalac AS1000^b^; and 2 swing loaders Mecalac AS900^c^) with vehicle cabins	Asphalt milling of areas in a residential area, in total 1,220 m^2^
B			Asphalt milling of a country road and a city road
C			Asphalt milling of city roads
D	The team consisted of 4 asphalt millers. The operator of the milling machine and the asphalt miller walking aside the milling machine to adjust the width of the milling, participated in the study	Milling machine with a cabin (Wirtgen W 200 Hi^d^)	Asphalt milling of a highway (a couple of km)

^a^EU Stage V/US EPA Tier 4f according to the vehicle manufacturer.

^b^EU Stage V/US EPA Tier 4 according to the vehicle manufacturer. Equipped with diesel particulate filter (DPF) and diesel oxidation catalyst (DOC).

^c^Stage III.

^d^EU Stage 4/US EPA Tier 4f according to the vehicle manufacturer.

**Table 2.** Description of the paving work performed during the field measurements.

Measurement day	Teams	Vehicles	Worksites	Used asphalt type^a^; approx. laying temperature (°C)	Used bitumen emulsion (BE) type
D	The team consisted of 9 asphalt pavers, and all participated in the study. The exposure measurements were performed over 2 workdays	Large paving machine (Vögele Super 1800-3i SJ^b^) with vehicle cabin Two large rollers (Dynapac CS1400 VI^c^; Bomag BW 174 AP 4f AM^b^) with cabins A vehicle that spread bitumen A material transfer vehicle (shuttle buggy; Roadtec SB-2500D) A small wheel loader with a cabin	Paving of a highway (a couple of km)	ABS16 70/100; 155	BE67 was used under the asphalt layer
E			Paving of a roundabout (1,370 m^2^) and a road (3,830 m^2^, 1.1 km) in a residential area. In total, 542 tons of asphalt	ABS16 45/80 AN7 PMB (polymer type) for the roundabout; 155 ABT16 70/100 for the road; 155	BE67 was used under the asphalt layer
F	The team consisted of 4 asphalt pavers, and 3 participated in the study.	Paving machine (Vögele Super 1803-3i^b^) with open cabin Roller (Hamm DV65 VO^d^) had a cabin	Paving of a small country road (1.7 km)	ABT11 100/150; 130	BE50R was used under the asphalt layer
G	The team consisted of 2 asphalt pavers and a truck driver, and all participated in the study.	Truck (VOLVO FH 8*4^e^) with a flexible screw conveyor (spreader) at the back laid the asphalt. Small roller (Bomag BW 100 ADM-5^b^) without a cabin A plate vibrator	Paving of 21 entrances to the small country road and 3 speed bumps on the road from day F	ABT11 100/150; 130	BE50R was sprayed on all the joints between the previously paved road
H	The team consisted of 5 asphalt pavers, and 3 participated in the study.	Paving machine (Vögele Super 1303-3i^b^) without cabin Roller (Hamm HD12^b^) without cabin	Paving of a city road (0.5 km) with 2 layers of asphalt	AG22 100/150 (carrier layer); 150 ABB16 70/100 (top layer); 150	No BE was used under the asphalt layer

^a^The initial letters represent the asphalt type, the number thereafter is the maximal stone size, and then the ratio shows the bitumen quality. ABS, stone-rich asphalt concrete; ABT, dense asphalt concrete; AG, asphalt gravel.

^b^EU Stage V/US EPA Tier 4f according to the vehicle manufacturer.

^c^EU Stage V/US EPA Tier 4 according to the vehicle manufacturer.

^d^EU Stage III A/US EPA Tier 3 according to the vehicle manufacturer.

^e^Euro 6.

#### Milling

Roads paved with asphalt were often milled to remove previous asphalt layer, for example before adding a new layer of asphalt on a road. For larger roads, milled asphalt was collected using larger milling machines and transported via a belt to a truck bed of a truck driving in front of the milling machine. For smaller roads and for minor areas, eg, on a bridge, smaller milling machines were used. The milled asphalt material was collected by a sweeper and loaded onto the truck bed of a nearby parked truck. At 3 of the worksites, the milling team consisted of a milling machine operator and an operator of the sweeper. Only at 1 worksite, a larger milling machine was used. The participating milling teams are described in Table 1.

#### Asphalt paving

Warm mix asphalt is manufactured and spread out at lower temperatures (100 to 140 °C) compared with conventional hot mix asphalt (150 to 190 °C) (Rubio et al. 2012). Warm mix asphalt (fossil-based) was used at 2 of the paving sites and at all other sites hot mix asphalt was used (Table 2). Asphalt was loaded in the front of the paving machines from trucks or from a material transfer vehicle (shuttle buggy). The paving machines, operated by asphalt pavers, were sometimes equipped with spray jet and sprayed bitumen mixture on the road before the asphalt was spread as a layer. The thickness and width of the asphalt layer were adjusted by the screed. Two pavers (screedmen), one at each side of the paving machine, adjusted the asphalt layer regarding width, corrected the edges of the asphalt layer manually, and smoothened out the asphalt with rakes. Thereafter, the asphalt layer was compressed by 1 or 2 rollers driven by roller operators. Pavers included operators of paving machines, rollers, and material transfer vehicles, screedmen, truck drivers, and team leaders. In total, 4 asphalt paving teams were included in the study, as described in Table 2.

### Study population

Non-smoking asphalt millers and pavers were invited to participate in the study. Prior to entry, all workers received both written and oral information about the study. The workers gave written informed consent in accordance with the Helsinki Declaration. The study was approved by the Swedish Ethical Review Authority (registration no. 2021-04138). A questionnaire was used to collect information on age, body mass index (BMI), gender, worktasks during the day of the measurement, consumption of barbecued food, snuff use, and having a smoking family member. In total, 26 male asphalt workers were included in the study. The mean age was 46 years, and the mean BMI was 30. Two pavers had consumed barbecued food within 24 h of participating in the study (Participants 12 and 16), and 6 participants were living with a partner who smoked (Participants 3, 4, 12, 17, 20, and 26). Twelve participants (1, 2, 4, 7, 9, 12, 13, 16, 17, and 19 to 21) used tobacco in the form of snuff.

### Field measurements

During the field experiments, exposure measurements of eBC, EC, OC, LDSA, PNC, particle size, PM_0.3_ of UFPs, NO_2_, and PAHs were carried out in the personal breathing zone (PBZ) of asphalt millers (*n* = 8) and asphalt pavers (*n* = 18) as full-shift measurements on average 9 h (min–max 5 to 12.5 h). All samplers and instrument inlets were placed in the PBZ outside working clothes (collarbone position). None of the workers used any respiratory protection. If the millers or pavers remained at the worksite during break or lunch, these periods were included in the sampling time.

During the field experiments, outdoor PAH measurements were performed with passive PAH samplers (described below) in 4 of the worksites (1 milling site and 3 paving sites) at least 200 m away from the worksite to assess background levels. The sampling height was 1 to 2 m. Three of the measurement locations were in urban areas and 1 was in a rural area, and there were no emission sources of PAH close to the locations.

### Direct-reading monitors

Portable aethalometers (model AE51, AethLabs, USA) measured real-time eBC mass concentrations, as a proxy for EC, with a flow rate of 100 ml min^−1^ and a time resolution of 30 s. A cyclone was used on the inlet with a cut-off diameter of 1.6 µm. The filter strip in the aethalometer was replaced before the start of the measurement, and after approx. 4-5 h of sampling. Portable aerosol monitors (Partector 2, Naneos, Switzerland) measured the real-time LDSA concentration of UFP in the size range 10 to 300 nm, with a time resolution of 1 s. The Partector 2 instrument also estimated the PNC, average particle size, and PM_0.3_.

The direct-reading monitors were carried by 21 of the 26 participants (7 millers and 14 pavers).

### Filter-based methods for active air sampling

#### Sampling of OC/EC

OC and EC were collected as time-integrated total dust fractions in the PBZ of 7 millers and 18 pavers. OC/EC samples were collected on double 25 mm quartz filters (SKC Inc., USA) mounted on support screens of stainless steel in open-face conductive 3-piece filter cassettes (SureSeal, SKC Inc.). Pumps (Apex2, Casella, UK) were used to provide sample flow rates set at 4.0 l min^−1^ according to NIOSH NMAM 5040 (NIOSH 2003). A flow meter (TSI Model 4100 Series, TSI Inc., USA) was used to regularly check the air flow rates. On each sampling day, a field blank sample for the OC/EC analysis was collected. The filters were stored at +5 °C until analysis.

#### Sampling of total dust

Total dust samples were collected as time-integrated mass fractions in the PBZ of 4 pavers. Samples were collected on 37 mm Teflon filters (Teflo, pore size 2 μm, Pall Corporation, NY, USA) mounted in open-face conductive 3-piece filter cassettes (SureSeal, SKC Inc.). Pumps (Apex2, Casella, UK) were used to provide a sample flow rate set of 4.0 l min^−1^. The air flow rates were regularly checked with a flow meter (TSI Model 4100 Series, TSI Inc., USA). The samples were analysed gravimetrically for determination of total dust. The limit of detection (LOD) was 0.05 mg sample^−1^.

### Passive air sampling methods

#### Sampling of PAHs

Polyurethane foam (PUF) samplers, which reliably accumulate both gaseous and particle-bound PAHs, were used to passively measure air concentrations of the sum of 16 US EPA PAHs of 8 millers and 18 pavers (Bohlin et al. 2010; Strandberg et al. 2018a, 2022; Gren et al. 2022; Lovén et al. 2024). The PUF sampler was placed in a tool holder on a badge plate together with the NO_2_ sampler in the PBZ. Four sets of duplicate sampling were performed, and 3 field blank samples were collected during the field measurements. The samples were stored at −20 °C until analysis.

#### Sampling of NO_2_

Passive samplers (IVL Swedish Environmental Research Institute, Gothenburg, Sweden) based on diffusion were used to measure airborne NO_2_ of 8 millers and 18 pavers (Ferm and Rodhe 1997; Ferm and Svanberg 1998). Average temperatures during the measurements ranged approximately between 8 and 20 °C. Three sets of duplicate sampling were performed, and 3 field blank samples were collected during the field measurements.

#### Self-administered air sampling of PAHs and NO_2_

The millers and pavers performed by themselves passive samplings of PAHs and NO_2_ at the following 2 workdays after the expert field measurement. At the first self-administrated occasion 5 millers and 18 pavers participated, and at the second occasion 5 millers and 17 pavers participated. They received oral and written information of how to perform the sampling and filled in details of sampling times, temperature, and worktask. The samplers were placed in the PBZs, and the sampling was performed during full workshifts.

### Wipe sampling of PAHs

Wipe sampling was performed similarly to previously used sampling techniques for PAHs in skin deposition and surface wipe studies (Keir et al. 2020; Banks et al. 2021; Lovén et al. 2024). Nonwoven swabs (7.5 × 7.5 cm, 2-ply, Mölnlycke Mesoft) were cleaned prior to use by extraction in dichloromethane (Supelco, HPLC grade) using ultrasonication, 3 × 15 min. The swabs were moistened with 1.0 ml of 2-propanol (Scharlau, Gradient HPLC grade) directly before wipe sampling. A fresh aliquot of 2-propanol was used each day to prevent contamination.

The palm of the dominant hand was wiped before and after the workshift. The size of the palms of the workers was measured using a ruler, and the wipe sampled area was calculated. Field blanks (*n* = 3) were performed as well as analysis of the 2-propanol used during sampling. Wipe sampling was performed using nitrile gloves, which were changed between the collection of each sample. Wipe sampling was also performed on 5 office workers (4 males, 1 female) without occupational PAH exposure as a control group. For additional information, see Supplementary material.

### Biological sampling of PAH metabolites

Spot urine samples from 25 participants were collected before and directly after the workshift. Urine samples were transported at 4 °C to the research laboratory, where they were aliquoted into 3 × 15 ml tubes, residues discarded, and stored at −20 °C prior to analysis.

### Chemical analysis

#### OC/EC samples

OC and EC were analysed at Ergonomics and Aerosol Technology, Lund University, with a thermal optical carbon analyser (DRI Model 2015 Series 2, Aerosol Magee Scientific, Slovenia) using the EUSAAR2 protocol (Cavalli et al. 2010). Seven field blank samples of OC/EC were included in the analysis, and all results were corrected by subtracting the mean value of the analysed field blank samples. The calculated concentration was then compared with 3 times the standard deviation of the field blanks to check if the value was above the LOD of the method. Applying this method, the LOD for EC and OC for 8 h sampling was 0.6 and 1.6 µg m^−3^, respectively.

#### NO_2_ samples

Analysis of NO_2_ was performed by IVL Swedish Environmental Research Institute in Gothenburg, Sweden, and the nitrite amount was analysed spectrophotometrically using flow injection analysis (Ferm and Rodhe 1997; Ferm and Svanberg 1998). Method LOD was 13 µg m^−3^ using 8 h sampling and measurement uncertainty was 10%. All 3 field blank samples of NO_2_ were below the LOD. Duplicate results are shown in Fig. S2.

#### PAH in air and wipe samples

PAHs were analysed at Occupational and Environmental Medicine, Region Skåne, according to a previously published method (Jorgensen et al. 2013; Strandberg et al. 2018b; Loive et al. 2024; Lovén et al. 2024). PUF samplers were extracted in dichloromethane using a Dionex ASE 350 Accelerated Solvent Extractor (Thermo Fisher Scientific Inc., USA) equipment. The wipe samples were extracted in dichloromethane by a Sonica ultrasonic extractor (Soltec, Italy). All samples were cleaned using a Pasteur pipette with some glass wool in the bottom and filled with 2 cm silica, and a small amount of sodium sulphate on the top, and eluted with dichloromethane. The target compounds, 16 US EPA priority PAHs (Zelinkova and Wenzl 2015), were separated on an Agilent 7010B GC/TQ triple mass spectrometer coupled to an Agilent 8890 GC system gas chromatograph (Agilent Technologies, Inc., USA). The LODs are presented in Supplementary material together with information about the 3 groups of PAHs (gaseous [L-PAH], intermediate [M-PAH], and particulate [H-PAH]) (Table S2). The detectability of PAH was high, and 93% to 100% of the samples were above LOD, see Table S2.

Concurrent sampling with passive PUF samplers and active pumped samplers, both gaseous and particulate fractions (37 mm Teflon filter [Teflo, pore size 2 μm, Pall Corporation, NY, USA]) and XAD-2 (Sorbent tube, SKC Inc., Eighty Four, PA, USA, flow rate of 2 l min^−1^), were performed to determine the uptake rates of PUF samplers in paving and milling work environments . This set of samplers was mounted on several stationary locations near the worksites as well as on work machines. The uptake factors were calculated and then used to determine the passive sampled PAH concentrations in this study. The results from the active PAH measurements and the PUF validation will be presented elsewhere.

#### PAH metabolites in urine

Urinary monohydroxylated metabolites of pyrene (1-OH-Pyr), phenanthrene (1-OH-Phe, 2-OH-Phe, 3-OH-Phe, and 4-OH-Phe), and fluorene (2-OH-Flu and 3-OH-Flu) (pyrene, phenanthrene, and fluorene abbreviated as sum 3 PAH) were measured by liquid chromatography coupled to tandem mass spectrometry (LC-MS/MS) as previously described (Alhamdow et al. 2020). Separation of the metabolites 2-OH-Phe, 3-OH-Phe, 2-OH-Flu, and 3-OH-Flu could not be achieved, so 2-OH-Phe, 3-OH-Phe as well as 2-OH-Flu and 3-OH-Flu are analysed as single peaks containing the 2 metabolites ∑ 2,3-OH-Phe and ∑ 2,3-OF-Flu, respectively. All PAH metabolite concentrations were adjusted to urinary creatinine values (µg/g Crea) (Carrieri et al. 2001). For details about the analysis and LOD, see Supplementary material.

### Statistics

Descriptive statistics for the exposure data is presented as geometric mean (GM), geometric standard deviation (GSD), and as minimum (min) and maximum (max) values. For statistical analysis, IBM SPSS Statistic 28 software for Windows was used. P–P plots showed that data were not normally distributed. Correlation between different exposure markers was analysed using bivariate Spearman correlation. Wilcoxon signed rank test was used to investigate if there was a difference between self-administrated and expert measurements, and for comparisons of exposure and biomonitoring data within each worker group. The Mann–Whitney *U* test was used for comparisons of exposures and biomonitoring data between asphalt pavers, millers, and non-occupationally exposed controls, respectively. The within-worker and between-worker variance were analysed as variance components with a general linear model on log-transformed data. The level of significance was set at *P* < 0.05. Values below the LOD were given the value of half the LOD.

## Results

### Air monitoring

Results from the personal exposure measurements of PNC, LDSA, PM_0.3_, average particle size, OC, EC, eBC, NO_2_, and 16 US EPA PAHs for asphalt millers and pavers are presented in Table 3. Individual exposure data are presented in Tables S3 to S5.

**Table 3.** Geometric mean (GM) values of the personal air, skin wipe, and urine samples for the 2 groups of asphalt workers.

	Exposure marker	Occupational groups GM (GSD) [min-max]	
		Asphalt millers (*n* = 8)	Asphalt pavers (*n* = 18)
Air samples	PNC^a^ cm^−3^	29,000 (4.0) [4,000 to 220,000]	31,000 (2.6) [8,200 to 320,000]
	LDSA^a^ µm^2^ cm^−3^	67 (2.3) [14 to 170]	80 (2.4) [19 to 350]
	PM_0.3_^a^ µg/m^3^	13 (3.6) [2.8 to 82]	38 (5.7) [1.2 to 420]
	Particle size^a^ nm	49 (1.5) [27 to 70]	55 (1.4) [33 to 91]
	OC µg m^−3^	40 (1.8) [13 to 82]	21 (2.5) [3.0 to 140]
	EC µg m^−3^	0.40 (3.8) [<0.1 to 1.6]	0.75 (3.2) [0.10 to 7.4]
	eBC^b^ µg m^−3^	0.56 (2.0) [0.16 to 1.1]	1.1 (3.3) [0.11 to 9.9]
	NO_2_ µg m^−3^	83 (3.6) [21 to 550]	31 (1.6) [17 to 99]
	Naphthalene ng m^−3^	29 (3.8) [6.1 to 370]	160 (4.1) [24 to 4,400]
	Benzo[a]pyrene ng m^−n^	0.39 (2.0) [0.16 to 1.1]	0.12 (5.1) [0.010 to 1.9]
	Sum L-PAH^c^ ng m^−3^	44 (3.0) [12 to 390]	270 (3.9) [48 to 6,500]
	Sum M-PAH^d^ ng m^−3^	3.1 (1.4) [1.9 to 4.9]	11 (3.5) [0.61 to 130]
	Sum H-PAH^e^ ng m^−3^	4.1 (1.8) [1.9 to 11]	2.9 (3.9) [0.69 to 22]
	Sum 3 PAH^f^ ng m^−3^	8.0 (1.2) [6.2 to 10]	70 (4.0) [12 to 1,500]
	16 US EPA PAH ng m^−3^	53 (2.7) [18 to 400]	290 (3.8) [53 to 6,600]
Skin wipes palm	Benzo[a]pyrene before work ng cm^−2^	0.0013 (2.1) [<0.0007 to 0.0028]	0.0031 (3.8) [<0.0007 to 0.027]
	Benzo[a]pyrene after work ng cm^−2^	0.0038 (2.1) [0.0012 to 0.0094]	0.0029 (2.7) [0.00077 to 0.026]
	Sum 3 PAH before work ng cm^−2^	0.030 (1.4) [0.020 to 0.052]	0.10 (1.7) [0.036 to 0.29]
	Sum 3 PAH after work ng cm^−2^	0.041 (1.4) [0.028 to 0.065]	0.14 (2.2) [0.040 to 0.99]
	16 US EPA PAH before work ng cm^−2^	0.061 (1.4) [0.035 to 0.11]	0.20 (2.0) [0.056 to 0.60]
	16 US EPA PAH after work ng cm^−2^	0.11 (1.4) [0.067 to 0.17]	0.25 (2.1) [0.068 to 1.5]
Urine samples	1-OH-Pyr before work µg/g Crea^g^	0.060 (4.4) [0.03 to 1.6]	0.058 (2.2) [0.010 to 0.24]
	1-OH-Pyr after work µg/g Crea	0.049 (2.1) [0.02 to 0.19]	0.085 (2.0) [0.030 to 0.23]
	Sum 3 OH-PAH^h^ before work µg/g Crea	0.36 (2.5) [0.29 to 2.8]	0.38 (1.7) [0.29 to 2.2]
	Sum 3 OH-PAH after work µg/g Crea	0.25 (2.0) [0.23 to 1.3]	0.56 (2.0) [0.38 to 3.9]

Individual exposure data are presented in Tables S3 to S5, where it also clearly can be seen which measurements were carried out on which participants.

^a^Seven asphalt millers and 13 asphalt pavers were equipped with Partector 2 instruments.

^b^Seven asphalt millers and 14 asphalt pavers were equipped with aethalometer instruments.

^c^Low molecular mass PAHs (>90% in gaseous phase).

^d^Intermediate (medium) PAHs (fractions in both gaseous and particulate phases).

^e^Particle bound, high molecular mass PAHs (>90% on particles).

^f^Sum of pyrene, fluorene and phenanthrene.

^g^Creatinine.

^h^Sum of metabolites of PAHs pyrene, fluorene and phenanthrene.

**Fig. 1.**
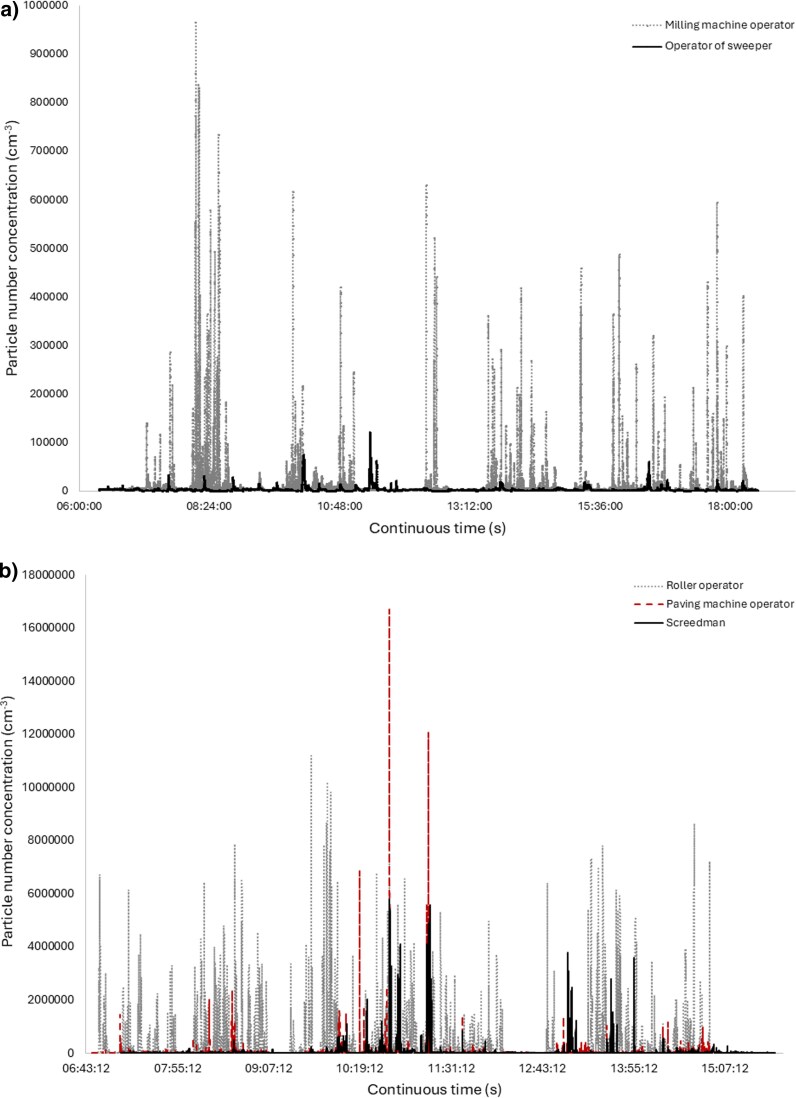
An example time series of PNC of ultrafine particles measured during a workshift with portable Partector 2 in the personal breathing zone of a) millers: a milling machine operator and an operator of a sweeper, and b) pavers: a paving machine operator, a roller operator, and an asphalt screedman. The size range was 10 to 300 nm with a time resolution of 1 s during 8-h workshifts. The average PNC exposure of the milling machine operator and the operator of the sweeper was 13,000 and 4,000 cm^−3^, respectively. The highest peaks origin from milling of asphalt. The corresponding PNC exposure for the paving machine operator, roller operator and screedman was 38,000, 320,000, and 71,000 cm^−3^. Compacting asphalt with roller generated many peaks over the workshift, while the paving machine generated a few high peaks.

The PNC exposures (GM) were at similar levels for asphalt millers (29,000 cm^−3^) and pavers (31,000 cm^−3^). The highest average PNC (320,000 cm^−3^) was measured for an operator of a roller without a vehicle cab. Among the asphalt millers, the highest exposure to PNC (220,000 cm^−3^) was measured in the PBZ of a sweeper operator. Example of time series of PNC can be seen for different milling (Fig. 1a) and paving (Fig. 1b) activities. The milling machine operator had both more and higher peak exposures to UFP measured as PNC compared with the operator of the sweeper. The roller operator was exposed to many peak exposures during the workshift compared with the paving machine operator and the screedman. However, the paving machine operator was exposed to the highest peaks of PNC.

LDSA of UFPs was also measured for the asphalt millers and pavers and was on average 67 and 80 µm^2^ cm^−3^, respectively. The average particle size ranged between 49 and 55 nm for both occupational groups. The asphalt pavers had almost 3 times higher exposure to PM_0.3_ mass (38 µg m^−3^) compared with the asphalt millers (13 µg m^−3^).

The occupational exposure to OC was on average 40 µg/m^3^ (GM) for the asphalt millers and 21 µg m^−3^ (GM) for the pavers. The highest exposure, 140 µg OC m^−3^, was measured in the PBZ of a paving machine operator. A comparison between OC, estimated organic mass, and PM_0.3_ mass is presented in Table S6.

The GM exposure to EC and eBC was almost twice as high for the asphalt pavers compared with the asphalt millers, with 0.75 (EC) and 1.1 (eBC) µg m^−3^ for the asphalt pavers and 0.40 (EC) and 0.56 (eBC) µg m^−3^ for the millers. The highest concentration of EC and eBC, 7.4 and 9.9 µg m^−3^, respectively, was measured in the PBZ of a team leader of the pavers who drove and worked from a passenger car that was partly idling.

The asphalt millers had more than 2 times higher NO_2_ exposure (83 µg m^−3^) compared with the asphalt pavers (31 µg m^−3^). The highest exposure to NO_2_, 550 µg m^−3^, was measured for an asphalt miller, who operated a sweeper (EU stage III) in a sparsely trafficked environment, indicating that the NO_2_ exposure mainly originated from the work machine.

Four exposure measurements of total dust were conducted on asphalt pavers, resulting in a GM of 52 µg m^−3^ (min–max 5.8 to 250 µg m^−3^).

The asphalt pavers had the highest exposure to 16 US EPA PAHs, 290 ng m^−3^ (GM) while the asphalt millers' exposure was at least 5 times lower than that of the asphalt pavers, 53 ng m^−3^ (GM). For the individual PAHs naphthalene, fluorene, phenanthrene, and pyrene, the asphalt pavers had higher GM exposures (3 to 14 times) compared with the asphalt millers, whereas for benzo[a]pyrene, the millers had more than 3 times higher exposure [0.39 ng m^−3^ (GM)] compared with the pavers [0.12 ng m^−3^ (GM)]. Furthermore, the levels of H-PAH were higher for millers compared with pavers, GM 4.1 and 2.9 ng/m^3^, respectively.

L-PAHs constituted the largest part of PAHs in all samples. For asphalt pavers, L-PAHs made up >90% and for 6 asphalt millers 70% to 85% of total PAHs. H-PAH and M-PAH, which are particle-bound and partly particle-bound, constituted about 15% to 35% of the total PAH content for the asphalt millers, but for asphalt pavers, this percentage was lower (<10%). Two asphalt millers differed from the other 6 in this occupational group. Naphthalene was the dominant PAH constituent for these 2 millers and made up about 90% of the total PAH content. Both these asphalt millers belonged to the same milling team. Naphthalene was dominant in the other samples as well but to a lower proportion, 40% to 75% for the asphalt pavers and for the other 6 millers, 35% to 65%. Other abundant PAHs, which constituted 5% to 22% of the total PAH content in all samples, were the L-PAHs phenanthrene, fluorene, and acenaphthene.

Statistical tests showed that there was only a significant difference between millers and pavers regarding the airborne exposure to 16 US EPA PAH (*P*-value = 0.004) including the individual agents such as benzo[a]pyrene (*P*-value = 0.024), naphthalene (*P*-value = 0.011), fluorene (*P*-value < 0.001), phenantrene (*P*-value < 0.001), pyrene (*P*-value = 0.008), and the sum of fluorene, phenanthrene, and pyrene (sum 3 PAH, *P*-value < 0.001).

In the outdoor background area of the 4 worksites, the sum of 16 US EPA PAH concentrations ranged between 3.0 and 30 ng/m^3^.

#### Self-administrated air sampling of 16 US EPA PAH and NO_2_

**Fig. 2.**
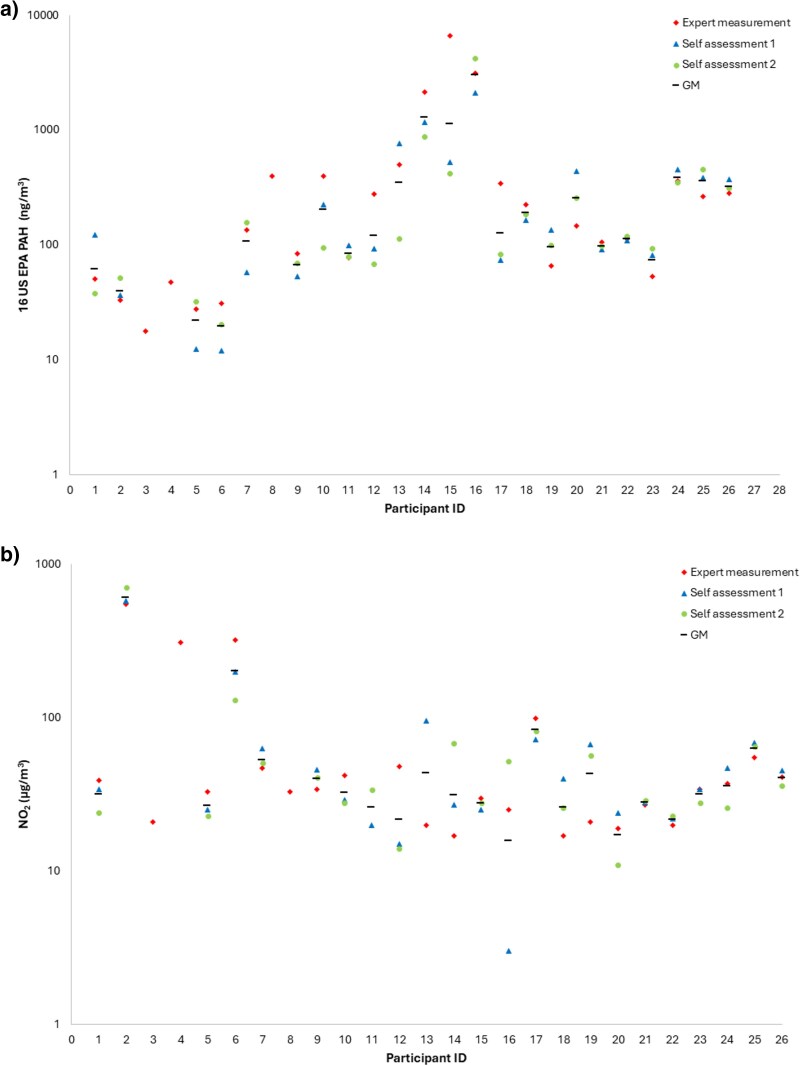
Repeated exposure measurements of a) 16 US EPA PAH and b) NO_2_ were performed by either occupational hygienists (expert measurement) or as self-administered sampling (self-assessment). Each data point represents 1 sample. Also, the GM exposure for each worker is shown. Workers with ID no. 1 to 8 were asphalt millers and 9 to 26 were asphalt pavers (values < LOD are presented as LOD/2). The *y* axis is presented with logarithmic scale.

The individual exposure to PAHs and NO_2_ obtained by self-administrated sampling is shown in Fig. 2. For millers, the calculations of the within-worker and between-worker variance of PAH and NO_2_ showed that the largest fraction of the total variance could be attributed to between-worker variability (63.4% and 96.3%), see Table 4. The within-worker variances of PAH and NO_2_ could be attributed to 36.6% and 3.7%, respectively, of the total variance. For pavers, the same pattern was seen for PAH with a between-worker variability of 74.8% while the within-worker variance was 25.2%. However, the opposite pattern was seen for NO_2_ exposure of pavers with a larger fraction of within-worker variance (71.4%) than the between-worker variance (28.6%).

**Table 4.** Variance components of personal exposure to PAH and NO_2_ within-worker and between-workers.

Exposure measure	Variance component	Miller	Paver
16 US EPA PAH	Within-worker (σ^2^_Within_)	0.245 (36.6%)	0.347 (25.2%)
	Between-worker (σ^2^_Between_)	0.425 (63.4%)	1.032 (74.8%)
NO_2_	Within-worker (σ^2^_Within_)	0.068 (3.7%)	0.260 (71.4%)
	Between-worker (σ^2^_Between_)	1.767 (96.3%)	0.104 (28.6%)

The self-administrated measured GM PAH exposure was 39 ng m^−3^ for the asphalt millers and 210 ng m^−3^ for the pavers. The overall (expert and self-administrated) GM PAH exposure was 45 and 240 ng m^−3^ for millers and pavers, respectively. The self-administrated and overall GM NO_2_ exposure was 83 and 33 µg m^−3^ for millers and pavers, respectively. The results from the first and second set of self-administrated sampling of NO_2_ did not statistically differ from the results of the expert measurement of NO_2_ (*P*-values = 0.52 and 0.79). There was no significant difference between the results of the expert measurements of 16 US EPA PAHs and the results from the first and second set of self-administrated sampling of 16 EPA PAHs (*P*-values = 0.47 and 0.51). Thus, the self-administrated sampling of NO_2_ and 16 US EPA PAHs reflects the same exposures as the expert measurements.

#### Correlation between exposure metrics in air

The correlation between the different exposure markers measured in the PBZ was analysed and can be seen in Table S7. Strong positive correlations were found between the following exposure metrics in air: 16 US EPA PAH versus PM_0.3_ (*P* = 0.002), EC versus eBC (*P* = 0.002), EC versus PNC (*P* < 0.001), LDSA versus PNC (*P* < 0.001), LDSA versus PM_0.3_ (*P* = 0.005), and sum 3 PAH versus PM_0.3_ (*P* = 0.005). There were also positive correlations between LDSA versus OC and EC, respectively.

### Skin wipe samples

Levels of pyrene, fluorene, phenanthrene (sum 3 PAH), the 16 US EPA PAHs, and benzo[a]pyrene on the palms of the dominant hands of workers before and after the workshift are presented in Table 3. The skin exposure to sum 3 PAH after the workshift correlated with the air levels of sum 3 PAH (*P* = 0.011) and to the urinary levels of metabolites of the 3 PAH (*P* = 0.027) when both occupational groups were analysed together. Asphalt pavers showed significantly higher levels of sum 3 PAH on the palm after the workshift compared with asphalt millers (*P* < 0.001). Asphalt millers showed higher levels of benzo[a]pyrene, albeit not significant, on their palm at the end of the workshift compared with pavers.

Most of the 16 US EPA PAH could be detected on the palm of millers and pavers both before and after the workshift. Control sampling performed on 5 office workers not occupationally exposed to PAH, showed a GM of sum 3 PAH of 0.051 ng cm^−2^ and of the 16 US EPA PAH of 0.099 ng cm^−2^. In the controls, a median of 8 out of the 16 US EPA PAH could be detected on the skin. Benzo[a]pyrene could be detected on the palms of 2 controls.

In the questionnaire, 21 participants reported using protective gloves during the workday, with 17 participants using textile and leather gloves and 1 participant using rubber and textile gloves. Three participants using protective gloves did not specify what kind. Eating or using snuff with bare hands and without prior hand wash was reported a median of 6 times during the workshift, indicating a risk of oral exposure to PAH. There was no difference in frequency of such hand-to-mouth contact between the millers and pavers.

### Monitoring of PAH in urine

**Fig. 3.**
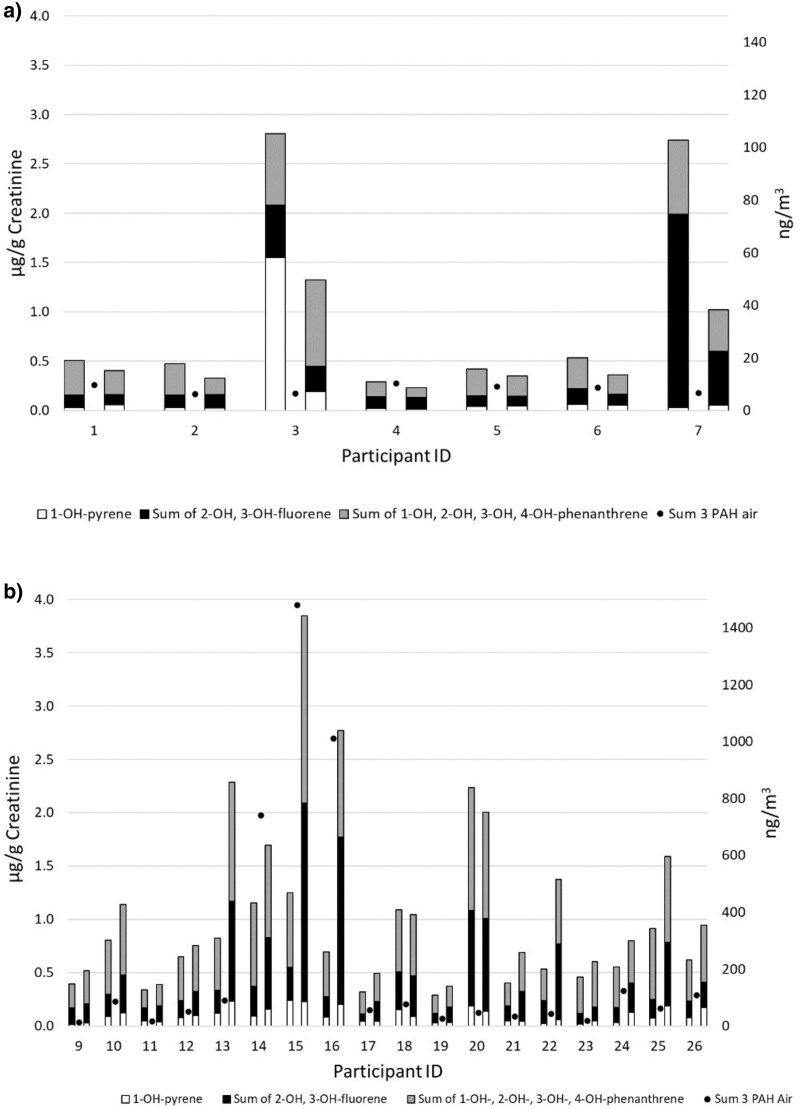
Urinary levels of metabolites of pyrene (white), fluorene (black), and phenanthrene (grey), ie sum 3 PAH, before and after the workshift in asphalt millers (a) and asphalt pavers (b). The individual airborne exposure to sum 3 PAH during the workshift is also shown.

Urinary levels of metabolites of 3 PAHs in asphalt millers and pavers are presented in Table 3 and Fig. 3. In asphalt pavers, there was a significant increase in urinary levels of 3 PAH metabolites after the workshift (*P* < 0.001). Millers had instead significantly lower levels of metabolites of 3 PAH in urine after the workshift compared with before the shift (*P* = 0.018). Urinary levels of PAH metabolites after the workshift were thus significantly higher in pavers compared with millers (*P* = 0.012).

## Discussion

### Airborne exposure to asphalt fumes, ultrafine particles and diesel exhaust

In this study, airborne exposures at asphalt milling and asphalt paving were measured using a multi-metric approach by novel exposure markers, which have not been used before to characterize asphalt fume exposure. Furthermore, repeated exposure measurements of PAH and NO_2_ were performed at up to 3 occasions on each participant, which characterized the exposures more accurately than previously reported in literature. As the between-worker variance mostly was high for these exposure markers, this indicates that the asphalt workers had differences in the exposures due to their different work tasks, and that their individual exposures were relatively uniform as the within-worker variance mostly was low.

To the best of the authors' knowledge, no studies of time-resolved personal exposure measured as LDSA of UFPs that deposit in the alveolar region during milling and paving of asphalt have previously been published in the literature. Only a few occupational exposures have been characterized by measuring LDSA, for example fire smoke at training of firefighters (Lovén et al. 2024) and soot at chimney sweeping (Moazami et al. 2024). During training of firefighters, low exposed personnel (observers and post-fire workers) had GM of 65 and 36 µm^2^ cm^−3^, respectively (Lovén et al. 2024). The chimney sweeps had an exposure to LDSA levels between 20 and 140 µm^2^ cm^−3^ (Moazami et al. 2024). Compared with the LDSA levels in this study, the asphalt millers (67 µm^2^ cm^−3^) and asphalt pavers (80 µm^2^ cm^−3^) were in the same range as low exposed personnel around fires and chimney sweeps.

The PNC of ultrafine particles in the PBZ was on average 29,000 cm^−3^ for millers and 31,000 cm^−3^ for pavers. In a previous Swedish study, PNC was measured during asphalt paving of a highway and the median concentration was then 34,000 cm^−3^ (Elihn et al. 2008). Compared with a US study, PNC were 43,000 cm^−3^ and 29,000 cm^−3^ for millers and pavers, respectively (Freund et al. 2012). Thus, the exposure to UFPs during asphalt paving is nearly at the same level today as a decade ago and PNC measured with pocket-sized monitors agreed surprisingly well with the levels in previous studies measured with condensation particle counters (CPC; P-Trak). As compared with the US study, the asphalt millers in Sweden were exposed to a lower average PNC. A typical PNC of UFPs in urban environments in Sweden was shown to be between approximately 10,000 and 20,000 cm^−3^ (Du et al. 2024).

The exposure to OC was on average 40 µg/m^3^ for asphalt pavers and 21 µg m^−3^ for the millers, which are in the same exposure range (42 µg m^−3^), that was measured for asphalt pavers in a previous Swedish study (Elihn et al. 2008). In a Norwegian study, GM OC air concentrations of 87 and 170 µg m^−3^ were measured during paving with warm and hot mix asphalt, respectively (Olsen et al. 2021). The main source of OC is the asphalt mix, and it has been suggested that measurements of OC could be a useful marker of exposure to air contaminants generated during asphalt paving (Elihn et al. 2008; Olsen et al. 2021).

The average air concentrations of 16 US EPA PAHs of asphalt pavers (GM 290 ng m^−3^) and asphalt millers (GM 53 ng m^−3^) in this study were lower than those measured in other European exposure studies of asphalt workers. For example, in Italian studies of asphalt pavers, the GM of 16 US EPA PAH was 3,500 ng m^−3^ (Fostinelli et al. 2018), and in a study by Cavallo et al. (2006), the arithmetic mean was 2,800 ng m^−3^. In a recent French study, asphalt millers had an exposure to gaseous PAHs of 7,800 ng m^−3^ (GM) during milling of old asphalt roads containing coal tar, and the particulate PAHs exposure was as GM 210 ng m^−3^ (Germin-Aizac et al. 2023). Milling of old asphalt roads containing coal tar could be associated with very high PAH exposures (up to 10 μg m^−3^). The PAH exposure of asphalt pavers in the same study was as GM of 1,000 (gaseous) and 11 (particulate) ng m^−3^, respectively. The highest 16 US EPA PAH exposures in this study were 6,600 ng m^−3^ for pavers and 400 ng m^−3^ for millers, respectively.

According to Germin-Aizac et al. (2023), the PAH content in asphalt was dominated by naphthalene, fluorene, and phenanthrene (2 to 3 aromatic rings), and the same PAH pattern was found in our study. Another French study of asphalt pavers reported that naphthalene represented 75% of the airborne PAHs (Deygout and Auburtin 2015), and that proportion is in the same range as we measured for the asphalt pavers (40% to 75% naphthalene), while asphalt millers had lower proportions (35% to 65%).

In one of the French studies, the personal exposure to benzo[a]pyrene during asphalt paving was in GM between 0.28 and 0.76 ng m^−3^ depending on the paving process (Germin-Aizac et al. 2023) and compared with this study, the GM for the asphalt pavers for benzo[a]pyrene was slightly lower (0.12 ng m^−3^). The Swedish occupational exposure limit (OEL) value for benzo[a]pyrene is set to 2,000 ng m^−3^ (Swedish Work Environment Authority 2023). All millers and pavers had exposures below this value, and the highest exposure was measured for a miller, who was exposed more than 1,000 times (1.9 ng m^−3^) lower than the OEL (not health-based). Typical concentrations of 16 US EPA PAHs, summer and winter, in urban sites in southern Sweden were shown to be 4.3 and 30 ng m^−3^ (Loive et al. 2024).

In this study, the asphalt millers had higher exposures to carcinogenic particulate PAH (H-PAH) compared with the asphalt pavers, and this pattern was seen in the air measurements and skin wipe sampling, but this was not seen with biological monitoring of urine metabolites.

In a previous Swedish study, EC was measured as a marker of diesel PM where the average concentration was 3.0 µg m^−3^ for asphalt pavers (Elihn et al. 2008). From Norway, it was reported that the GM air EC concentrations were measured to 2 and 3 µg m^−3^ during paving with warm mix asphalt and hot mix asphalt, respectively (Olsen et al. 2021). The corresponding average EC levels in this study were for asphalt pavers and millers 0.75 and 0.40 µg m^−3^, respectively, and the highest exposure was measured for a paver (7.4 µg/m^3^). Thus, today the occupational exposure to EC from diesel exhausts from traffic and work machines seems to be lower compared with the reported levels in 2008, probably due to sharpened emission legislation for diesel exhaust and working machines in combination with electrified vehicles. However, estimates of the lifetime mortality of lung cancer from 45 years of occupational exposure to diesel exhaust is 17 excess deaths per 10,000 for exposure at 1 μg EC m^−3^, and 200 excess deaths per 10,000 for exposure at 10 μg EC m^−3^ (Vermeulen et al. 2014). The Swedish OEL for diesel exhaust measured as EC is set to 50 µg m^−3^, and this is not health-based (Directive (EU) 2019/130, 2019; Swedish Work Environment Authority 2023).

Furthermore, NO_2_ concentration reflects the occupational exposure to diesel exhaust from the workers' own work machines as well as from the surrounding traffic. The operators who drove sweepers had the highest exposure to NO_2_ (310 to 550 μg m^−3^), but their EC exposures were low (up to 1.6 µg m^−3^). These operators drove back and forth as they swept up the milled asphalt and thus repetitively drove into their own diesel exhausts. These results indicate that the exhaust after treatment system is inefficient in the sweepers even if 1 of the sweepers adhere to the latest emission legislation (EU Stage V). This can be explained by the relatively weak emission regulation for NOx below 56 kW in Euro Stage V (Dieselnet 2025). In the 19 to 56 kW range, PN regulation is strict, commonly requiring a diesel oxidation catalyst (DOC) and diesel particulate filter (DPF) combination. A combination of DOC and DPF converts some of the NO emissions to NO_2_, which may increase the NO_2_ emissions and eventually NO_2_ exposure, even if the NOx (NO + NO_2_) emissions are moderate.

It would be beneficial to replace diesel-powered vehicles with electrical vehicles. The milling machine operators had a much lower exposure to NO_2_ (21 to 47 μg m^−3^). In general, the asphalt pavers also had a low exposure to NO_2_. Direct-reading monitors were also used to measure eBC as a proxy for EC. The concentrations of EC and eBC were in similar ranges for both millers and pavers. To the best of the authors' knowledge, no studies of time-resolved personal exposure to eBC during milling and paving of asphalt have previously been published in the literature. In this study, the eBC/EC ratio was on average the same for both occupational groups, 1.4 for pavers and 1.5 for millers, respectively. These 2 metrics showed a significant correlation (*r* = 0.64, *P* = 0.002, Table S7) although different particles size fractions and measurement techniques were used.

By using self-assessed exposure measurements of PAH and NO_2_ we have exposure data for 3 workshifts for most of the participants, which strengthen the exposure characterization. This study design has not previously been applied for exposure measurements of millers and pavers, and it has thus been possible to receive accurate exposure data for more than 1 workday without time-consuming field measurements. From previous studies, we have positive experience of self-assessments of exposure, and that exposure data did not statistically differ from the ones collected by experts (occupational hygienists) (Hedmer et al. 2017; Gren et al. 2022; Lovén et al. 2024). For most of the participants, the concentrations were in the same range for all 3 days, which indicates a low within-worker variation of the exposure (Fig. 2, Table 4). It was also possible to see the between-worker variation for both within and between the occupational groups. It was a significant difference between millers and pavers exposure to 16 US EPA PAH.

### Dermal exposure to PAHs

Skin exposure to PAH, measured as 3 PAH and as 16 US EPA PAH, was found to correlate with air levels of these PAH, indicating that the skin can be an important exposure route to PAH in asphalt workers. For PAH of lower molecular weight (L-PAH and M-PAH), previous studies have found the same correlation between skin exposure and air levels (McClean et al. 2004; Vaananen et al. 2005). However, in the present study, there was no correlation between air- and skin levels of benzo[a]pyrene in either asphalt millers or asphalt pavers, indicating other routes of exposure. Furthermore, millers were highly exposed to benzo[a]pyrene compared with PAH of lower molecular weight. This is confirmed by previous studies, where PAH-H was found to be dominant in skin exposure to PAH (Herrick et al. 2007).

The measured levels of PAH on the skin at the end of the workshift are not considered to be equal to the total skin exposure during the workshift, due to skin absorption and decontamination of PAH during work, and to the sampling method. Wipe sampling as a surface sampling method was tested, and the experiments are described in Supplementary material. Wipe sampling in a porous material, eg skin, could be more difficult but can still provide good estimates of PAH exposure. Isopropanol was chosen as sampling solvent as it interferes less with the analysis method compared with sunflower oil, which is a commonly used alternative for wipe sampling (Vaananen et al. 2005). The results can therefore not be directly compared with levels reported in the literature.

A further exposure route to PAH resulting from skin exposure is hand-to-mouth oral exposure. In the questionnaire, 12 out of 25 participants used snuff and 17 had used snuff or eaten food they had held with unwashed bare hands a median of 6 times during the workshift. This could contribute to oral exposure to PAH. The asphalt pavers had no means of washing their hands and in many cases had lunch while continuing to work. This means of exposure could quite easily be decreased by introducing wet wipes or similar means of hand sanitation.

### Systemic exposure to PAHs

Investigations of urinary PAH metabolites in young Swedish adults, using the same analytical method as in the present study, have found median urinary concentrations of 0.057 µg/g Crea of 1-OH-Pyr and 0.59 µg/g Crea of the sum of 1-OH-Pyr, 2- and 3-OH-Flu, and 1-, 2-, 3-, and 4-OH-Phe (sum of 3 OH-PAH) (Alhamdow et al. 2021). Mean urinary levels of 1-OH-Pyr as well as the sum of 3 OH-PAH before the workshift among the asphalt millers were in the same range as the median levels in occupationally unexposed adults reported by Alhamdow et al. (2021). However, the asphalt millers showed higher urinary levels of OH-PAH before the workshift compared with after, with participants 3 and 7 showing high levels before the workshift. None of these participants had consumed barbecued food prior to the sampling. Participant 3 was, however, living with a partner who smoked. Participant no. 7 was working during a workshift where paving was performed in parallel with the milling; thus, the exposure for this miller could have been influenced by the paving performed close to the milling work. In many asphalt pavers, urinary OH-PAH levels were higher than the median levels in occupationally unexposed adults (Alhamdow et al. 2021) and increased after the workshift. Although the pavers were exposed to higher general air levels of PAH, the asphalt millers seem to be exposed to higher levels of the more carcinogenic H-PAH. This was also reflected in the skin exposure. The metabolites of PAH investigated in the biomonitoring do not reflect this, as the parent PAH to these metabolites are L- and M-PAH.

### Limitations in the study

The study had an exploratory design with a relatively low number of participants (especially millers) in each investigated group, limited by what was practically and economically feasible. The millers mainly worked in small teams with 2 workers and all non-smoking milling teams in the south of Sweden were recruited in the study.

PNC measured with newer and smaller techniques such as Partector 2 are less reliable compared with results from more advanced direct-reading instruments (eg CPC).

All measurements and sampling were performed outdoors, which entails more difficult conditions compared with indoor environments due to, eg, air turbulence and wind speed, which are parameters that could affect the performance of passive samplers.

## Conclusion

This study showed that asphalt millers and pavers were exposed to airborne PAHs, UFPs, OC, and diesel exhaust. Exposure was measured using a multi-metric approach by novel exposure markers, based on both online techniques and offline methods as well as active and passive sampling, which have not been used before to characterize asphalt fume exposure. With a study design that involved repeated exposure measurements on consecutive workshifts for each participant, more accurate exposure characterization and assessment could be obtained. By using portable aerosol monitors, valuable exposure data of novel metrics were obtained. Operators of, eg, rollers and milling machines were exposed to many peak exposures during the workshift compared with other asphalt workers at the worksites. The workplace exposure to diesel exhaust was in general lower today compared with previous studies; however, the sweeper operators in the milling teams were highly exposed to NO_2_ as they repeatedly drove into their own exhaust. Their exposure could be reduced if electrical sweepers were used instead of diesel-powered. Millers were exposed to higher levels of carcinogenic particulate PAH (H-PAH) compared with the levels of gaseous (L-PAH) and intermediate PAHs (M-PAH). As biomonitoring generally is performed by measurements of metabolites of L-PAH and M-PAH, this exposure could not be detected using biomonitoring. Air and skin exposure measurements were vital in order to detect this exposure as both inhalation and skin exposure are important exposure routes of PAHs. A possible exposure route for PAH was identified as oral exposure through hand-to-mouth contact (oral exposure), since the asphalt workers lacked the opportunity to wash hands before breaks. Using wet wipes before meals or snuff use would be a simple way to decrease exposure via this pathway.

## Supplementary Material

wxaf078_Supplementary_Data

## Data Availability

The data underlying this article will be shared on reasonable request to the corresponding author.
