# Supplementary material for: Characterization of occupational exposure to air pollutants during asphalt milling and paving
Source: Ann Work Expo Health. 2025 Nov 20;70(2):wxaf078. doi: 10.1093/annweh/wxaf078 (PMC13016829; doi:10.1093/annweh/wxaf078)
Supplement: wxaf078_Supplementary_Data [file wxaf078_supplementary_data.pdf]

SUPPLEMENTARY MATERIAL

Characterization of occupational exposure to air pollutants during asphalt milling and asphalt paving

Maria Hedmer[1,2*], Karin Lovén[1,2], Johannes Rex[3], Carina Nilsson[1,2], Merve Polat[4,5], Jakob K. Nøjgaard[4,5], Joakim Pagels[3], Bo Strandberg[1,2], Lina Hagvall[1,2]

[1]Department of Occupational and Environmental Medicine, Skåne University Hospital, SE-22381 Lund, Sweden

[2]Division of Occupational and Environmental Medicine, Department of Laboratory Medicine, Lund University, SE-22100 Lund, Sweden

[3]Ergonomics and Aerosol Technology, LTH, Lund University, SE-22100 Lund, Sweden

[4]National Research Centre for the Working Environment, DK-2100 Copenhagen, Denmark

[5]Department of Chemistry, University of Copenhagen, DK-2100 Copenhagen, Denmark

Material and methods

Workplace measurements

The occurring meteorological conditions during the workplace measurements are presented in Table S1.

Table S1. Meteorological conditions during the workplace measurements.

Measurement day	Meteorological conditions	
	**Average measured temperature (°C)**	**Weather**
A	14	Cloudy weather and wind
B	20	Clear and sunny weather
C	21	Clear and sunny weather
D	11	Cloudy weather and rain showers
E	14	Sunny weather
F	13	Partly clear weather
G	8	Partly clear weather
H	13	Partly clear weather and a little wind

Chemicals and Reagents

All adsorbents, silica gel 60 (Merck, Darmstadt, Germany) and sodium sulphate (Merck, Darmstadt, Germany) were cleaned by thermal treatment at 450°C and activated at 100°C before use. The solvents dichloromethane and n-hexane were of glass distilled quality (Merck, Darmstadt, Germany) and 2-propanol of HPLC grade (Scharlau, Gradient HPLC grade). A deuterated internal standard mixture (1 ng µL^{-1}) containing the 16 U.S. Environmental Protection Agency (US-EPA) priority PAHs (Dr. Ehrenstorfer (Augsburg, Germany) were used. A native mixture containing 16 US EPA PAHs (Dr. Ehrenstorfer, Augsburg, Germany), all at 1 ng L^{-1}, was used for detection and quantification of target compounds. Octachlorornaphthalene (OCN) (Ultra Scientific, North Kingstown, RI, USA) (1 ng L^{-1}) was used as recovery determination standard (RS). Quality controls (QCs) (SRM 1649b) were purchased from the US National Institute of Standards and Technology (NIST) (Gaithersburg, MD, USA).

PAH air sampling method

The passive polyurethane foam (PUF) air sampler (length: 10 cm, diameter: 2.2 cm), with a total surface area and density of 77 cm^2 and 0.030 g cm^{-3} (Klaus Ziemer GmbH, Germany) were used as personal samplers. This sampler design has been calibrated for uptake-rates, both as personal and stationary sampler for both gaseous and particulate associated PAH at an alloy industry and a coke plant (Bohlin et al., 2010) and, moreover, as a passive personal sampler to estimate the occupational exposure of firefighters and police forensic specialists (Strandberg et al., 2018). To assess the performance and ensure the uptake-rates for PUF samplers in paving and milling work environments, concurrent sampling with the passive PUF sampler and active pumped sampling, both gaseous and particulate fractions were performed to determine the

uptake-rates of PUF samplers in the present work environments. Several stationary locations near the work environments as well as this set of sampler equipment mounted on work machines near the workers were used for this purpose. The uptake factors were calculated and then used to determine the PAH levels from the passive sampling in the personal breathing zone in the study. The results from the active pump measurements and from the PUF sampler validation in these working environments for many PAH compounds including alkylated, nitrated and oxidized PAH derivatives will be presented elsewhere (Polat et al., manuscript in prep.; Strandberg et al., manuscript in prep.).

PAH wipe sampling method

The swabs used in the skin deposition and surface wipe studies were nonwoven swabs (7.5×7.5 cm, 2-ply, Mölnlycke Mesoft). Before use the swabs were cleaned three times for 15 min by extraction in dichloromethane (Merck, Darmstadt, Germany). After each purification process the solvent was drained off and new solvent was added. The swabs were then allowed to dry in a fume hood for 30 min and then wrapped in aluminium foil, 10 in each, and placed in gas-tight bags. Directly before use the swabs were moistened with 1.0 mL of 2-propanol (Scharlau, Gradient HPLC grade). The skin surface sampling of the palm was done by moving the swab up and down three times vertically and then up and down three times horizontally. The swab was then rolled up with the swabbed surface inwards and placed in 10 mL amber vials and the caps were screwed on.

The wipe sampling method with subsequent extraction and purification was tested with two experiments: In the first experiment 40, 100 and 200 uL of the native mixture were spiked into three vials containing 5 mL isopropanol, respectively. This corresponds to 40, 100 and 200 ng for each PAH. Each solution was applied to a well-cleaned laboratory bench surface (approx. 10 × 20 cm) and allowed to dry. After one hour, the bench surface was wiped as described above.

In the second experiment, approximately 0.5, 1 and 3 mg of SRM 1649b were weighed onto cut-out 37-mm Teflon filters (Pall Corporation, NY, USA). The weighed sample was smeared over a 10 × 20 cm area on a well-cleaned laboratory bench surface. The surface was wiped as described above. The filter was also included in the wipe since some particles remained on the filter.

In the first experiment, recovery was acceptable for all PAHs (>85%), within the mass range 40-200 ng except for naphthalene. The recovery for naphthalene was however somewhat lower, around 50-70%. The reason may be attributed to the higher volatility for this compound and that after one hour it has already started to evaporate or that this PAH is more difficult to sample with the wipe method. The results of the second experiment for 11 particulate US-EPA PAHs the results were within 25% of the certified levels (Figure S1).

We judged the results of the two experiments acceptable but would like to emphasize that wipe sampling in a porous material such as skin may be more difficult but can still provide a good estimate of PAH levels on the skin surface. Furthermore, the results confirms that the extraction and purification method for sampled PAH substances works very well for this sampling method.

Analysis of polycyclic aromatic hydrocarbons

All samples, before extraction, were spiked with 40 µL of the deuterated internal standard mixture. The extraction equipment used for the PUF samples was a Dionex ASE 350 Accelerated Solvent Extractor equipment (Thermo Fisher Scientific, Inc. MA, USA). The PUFs were placed in extraction cells of 60 mL, filled with 4 g of silica in the bottom and then extracted using dichloromethane as solvent. The extraction was performed at 100°C and three static time

cycles at 5 min each. Following extraction, all samples were evaporated to a final volume of approx. 4-6 mL and quantitatively transferred to 10 mL amber vials. The eluate fraction was evaporated under nitrogen flow to a final volume of approx. 1 mL.

The wipe samples were extracted three times in approx. 7 mL dichloromethane by a Sonica ultrasonic extractor (Soltec, Italy). After each extraction, the sample was transferred to a 25 mL amber vial. The elute was evaporated under nitrogen flow until approx. 2 mL left, transferred quantitatively to a 4 mL vial and the solvent was evaporated down to approx.1 mL.

All samples (PUF and wipe) were purified using a Pasteur pipet filled with 2 cm silica (SiO_2) and some sodium sulphate on top. The elute was evaporated under nitrogen flow until to approx. 200 µL. Samples were transferred to GC glass insert vials (Agilent Technologies) and 40 µL of RDS was added and samples were reduced to a small volume (approx. 30-40 µL) for analysis.

Target compounds were separated on an Agilent 8890 GC System gas chromatograph coupled to an Agilent 7010B GC/TQ triple mass spectrometer (MS). Samples 0.3 µL were injected using an Agilent 7693A autosampler unit. The capillary column used was a DB-5MS (30 m × 0.25 mm, 0.25 µm, Agilent Technologies). Helium was the carrier gas at a flow rate of 1.0 mL/min. The temperature program was as follows: initial temperature 50° C for 3 min; ramp at 10°C/min to 180°C and held for 5 min; ramp at 3°C/min to 300°C and held for 20 min; injection at a transfer line temperature of 270°C. Electron impact ionization (EI) was performed for all target compounds at 70 eV energy and at a 280°C ion source temperature. The MS was operated in multiple reaction monitoring mode (MRM).

Table S2. Information about the PAH-groups and which individual 16 EPA PAH belongs to which group. The limit of detection (LOD), each sampler type (air: PUF and skin: wipe), instrumental detection limit (IDL), and detectability for each compound are also given.

Group	Phase	PAH	LOD PUF (ng m^{-3})	LOD wipe (ng m^{-3})	IDL (fg)	Percentage above LOD
L-PAH (low molecular mass PAHs)	Gaseous	Naphthalene	<1.1	<0.0057	30	100
		Acenaphthylene	<0.24	<0.00065	65	100
		Acenaphthene	<0.15	<0.00030	65	100
		Fluorene	<0.090	<0.00057	65	100
		Phenanthrene	<0.11	<0.00053	65	100
		Anthracene	<0.030	<0.00090	65	96
M-PAH (intermediate molecular mass PAHs)	Both gaseous and particulate	Fluoranthene	<0.010	<0.0012	65	100
		Pyrene	<0.010	<0.0026	65	100
H-PAH (high molecular mass PAHs)	Particulate	Benzo[a]anthracene	<0.010	<0.0014	65	100
		Chrysene	<0.030	<0.0019	65	96
		Benzo[b]fluoranthene	<0.030	<0.0011	500	100
		Benzo[k]fluoranthene	<0.010	<0.0011	500	100
		Benzo[a]pyrene	<0.010	<0.00070	500	100
		Indeno[1,2,3-c,d] pyrene	<0.010	<0.00055	500	96
		Dibenzo[a,h]anthrace ne	<0.010	<0.00056	500	93
		Benzo[g,h,i]perylene	<0.010	<0.00055	500	96

PAHs refer to the sum of the 16 US Environmental Protection Agency (US-EPA) priority PAHs (Zelinkova and Wenzl, 2015), L-PAHs refer to low molecular mass PAHs (>90% in gaseous phase), M-PAHs refer to intermediate molecular mass PAHs (fractions in both gaseous and particulate phases), H-PAHs refer to particulate phase, high molecular mass PAHs (>90% on particles), and sum 3 PAH refers to sum of pyrene, fluorene and phenanthrene. Table S2 presents which individual PAH belongs to L-, M- and H-PAHs.

Quality assurance/Quality control

PAH

Consistent recovery (55-115%) was obtained for internal standard compounds that were added to and used for correction of the samples.

PUF and wipe (n=3) field blanks were analysed in parallel with the samples. Some minor residues of some 2-4 ringed PAHs occurred. All PAH results were corrected to the blanks. The limit of detection (LOD) was calculated as three times the standard deviation of the blanks. The LOD for each compound is given in Table S2. The wipe sampling evaluation test using the SRM 1649b urban dust), and three quality control (QC) samples were used as QCs to ensure the analysis. The measured levels of 11 PAHs (Figure S1a) were for most part, within 25% of the certified levels.

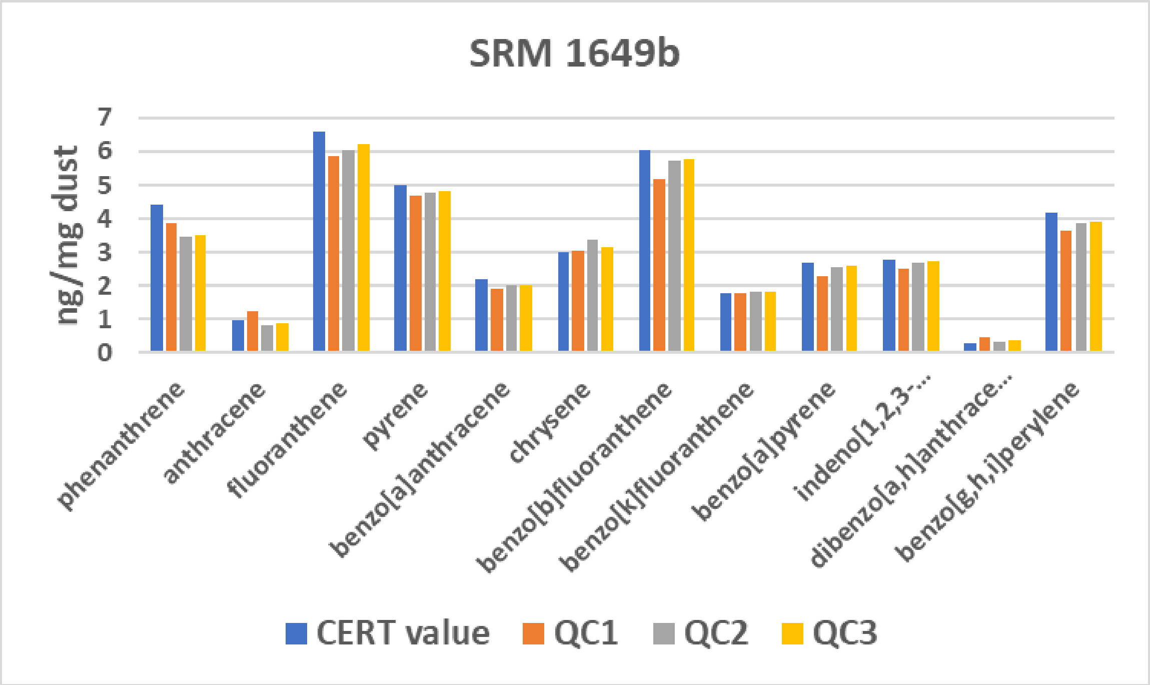

Figure S1. Results of three quality control samples (QCs) (NIST SRM 1649b) compared to published certified values of 11 US-EPA PAHs. Results are from the evaluation of the wipe method.

Four duplicate samplings, two for each miller and paver were carried out (Figure S2a). There were good agreement agreements between the duplicates; the deviations from their means were about 1-35%. A somewhat higher deviation (35-70%) were observed for some of the particulate PAHs in the sample of Miller 5, which is likely due to a greater uncertainty in the determination as the PAH levels were low and close to the LOD.

Nitrogen dioxide

Duplicate sampling of nitrogen dioxide (NO_2) was performed in three of the measurements. Two NO_2 samplers were placed on the same participant, on one asphalt miller and two asphalt pavers (Figure S2b). The results showed that there was good agreement between the duplicates.

a)

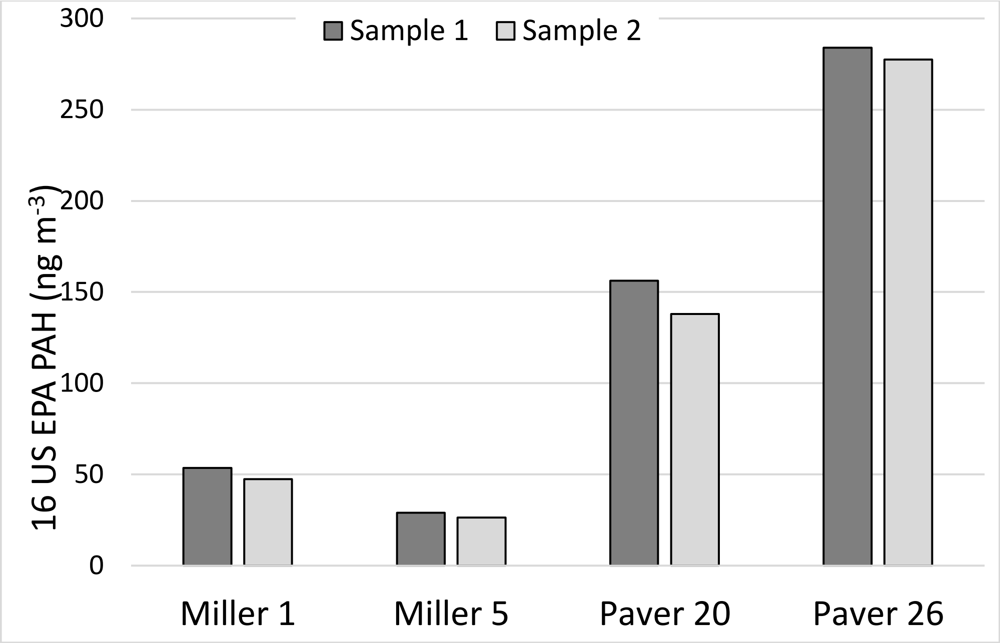

b)

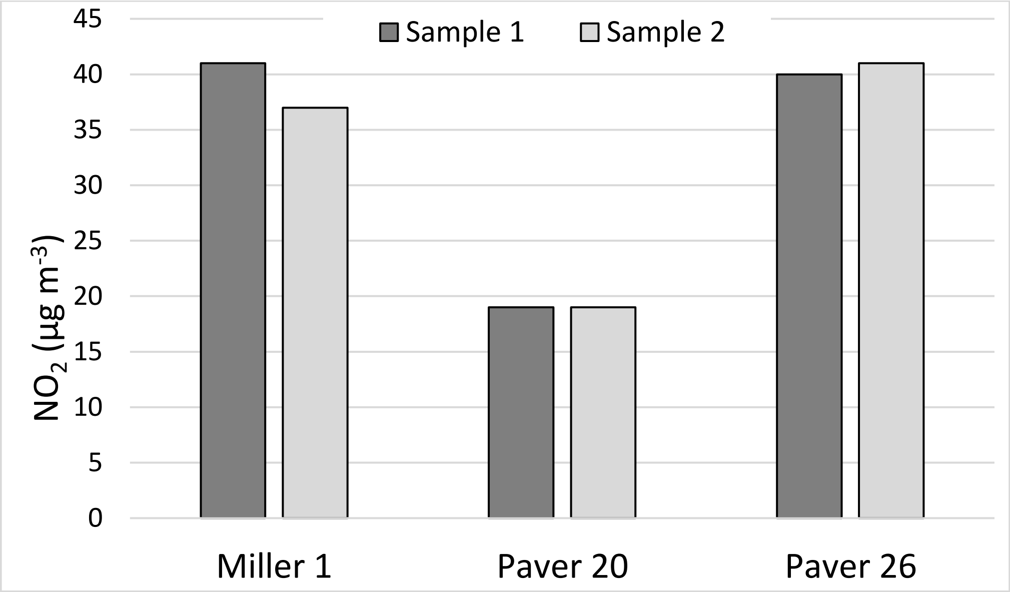

Figure S2. Duplicates of a) 16 US EPA PAHs and b) NO_2 were sampled on four and three workers, respectively, asphalt millers and asphalt pavers. The results showed that there were good agreements between the duplicates.

Chemical analysis of PAH metabolites in urine

The separation was achieved using a Phenomenex LUNA 2,5µm C18-HST 100 × 2.1 mm, column (2.6 µm, 100 Å, 2.1 mm i.d. × 100 mm, Phenomenex, Torrance, California, USA) maintained at 40°C. The mobile phase was water (A) and methanol/acetonitrile (1/1) (B). The gradient was linearly increased from 5% B to 95% B within 8 min and held there for 3 min. Samples were prepared and run in duplicates and the average values were used. LOD values were calculated from chemical blanks and was 0.02 n OH-Flu g ml^{-1} for 1-OH-PYR, 0.01 ng ml^{-1} for 1-OH-Phe, 0.006 ng ml^{-1} for ∑ 2,3-OH-Phe, 0.007 ng ml^{-1} for 4-OH-Phe and 0.006 ng ml^{-1} for ∑ 2,3-OH-Flu.

Results

Table S3-S5 shows the individual exposure data for asphalt millers and asphalt pavers.

Estimation of organic mass

Organic carbon (OC) was sampled with filter-based methods and analyzed with a thermo-optical method. The OC-method assesses the mass of carbon atoms of organic carbon compounds. However, the sampled organic compounds also consist of atoms of other elements such as hydrogen (H), nitrogen (N), sulphur (S), oxygen (O), phosphor (P) and others. To estimate the organic matter (OM) of the sampled organic compound from OC concentrations a multiplying factor could be used (Turpin and Lim, 2001). The factor of 1.2 g cm^{-3} used in this study was estimated from urban data by Turpin and Lim as most of the air measurements were performed in urban environments (2001). We also made an assumption that all OC was alkanes and aromatic compounds composed of mainly C and H. The estimations of OM in this study can be seen in Table S6.

Correlation analysis of exposure metrics

The results from the correlation analysis of the exposure markers measured in the personal breathing zone measurements are presented in Table S7.

References

Bohlin P, Jones KC, Levin J-O, Lindahl R. Strandberg B. Field evaluation of a personal passive sampler for PAH exposure in workplaces. Journal of Environmental Monitoring 2010; 12; 1437-44.

Polat M, Rex J, Strandberg B, Nøjgaard J, Lovén K, Hagvall L, Hedmer M, Johnson M, Pagels J. Characterization of particulate emissions during asphalt paving in southern Sweden. Manuscript.

Strandberg B, Lovén K, Polat M, Rex J, Nøjgaard J, Pagels J, Hedmer M, Hagvall L. The use of polyurethane foam passive air sampler (PUF) in occupational exposure studies of PAHs and their nitro- and oxy- derivative compounds during asphalt paving and milling. Manuscript.

Strandberg B, Julander A, Sjöström M, Lewné M, Akdeva HK, Bigert C. Evaluation of polyurethane foam passive air sampler (PUF) as a tool for occupational PAH measurements. Chemosphere. 2018; 190: 35-42.

Turpin BJ & Lim H-J. Species Contributions to PM2.5 Mass Concentrations: Revisiting Common Assumptions for Estimating Organic Mass. Aerosol Science and Technology. 2001; 35: 602–10.

Zelinkova, Z., Wenzl, T. The occurrence of 16 EPA PAHs in food–a review. Polycycl. Aromat. Comp. 2015; 35, 248–84.

Table S3. Individual average values of a) the personal air and b) urine samples of the asphalt millers. Standard deviation and the maximum value are also shown for the direct-reading measurements of eBC, LDSA, PNC, particle size, and $PM_{0.3}$. It can also be seen which measurements were carried out on which participant.

a)

		Asphalt millers							
	**Participant ID**	***1***	***2***	***3***	***4***	***5***	***6***	***7***	***8***
**Air samples**	**Measurement day**	A		B		C		D	
	**Work task**	Milling machine operator	Operator of sweeper	Milling machine operator	Operator of sweeper	Milling machine operator	Operator of sweeper	Milling machine operator	Walk aside the milling machine, adjust the milling width
	**Sampling time** min	706	727	472	460	585	568	304	362
	**eBC** µg m^{-3} (STD) [max]	0.69 (1.4) [12]	0.74 (12) [460]	0.36 (1.1) [14]	1.0 (1.2) [9.5]	0.52 (2.2) [52]	0.16 (0.50) [11]	1.1 (4.6) [87]	-[a]
	**EC** µg m^{-3}	0.54	0.33	<0.20	1.3	<0.10	1.6	1.0	-
	**OC** µg m^{-3}	32	13	36	59	46	82	45	-
	**NO_2** µg m^{-3}	39	550	21	310	33	320	47	33
	**LDSA** µg m^{-3} (STD) [max]	50 (200) [4660]	14 (12) [170]	73 (290) [4900]	91 (290) [11200]	62 (150) [10250]	124 (150) [1460]	170 (270) [2010]	-
	**PNC** cm^{-3} (STD) [max]	13000 (42000) [970000]	4000 (6100) [120000]	19000 (61000) [990000]	130000 (410000) [10000000]	17000 (49000) [2600000]	220000 (300000) [2900000]	38000 (63000) [1200000]	-
	**Particle size** nm (STD) [max]	60 (13) [130]	70 (21) [700]	54 (13) [150]	27 (14) [92]	63 (15) [150]	27 (25) [150]	63 (24) [230]	-
	**$PM_{0.3}$** µg m^{-3} (STD) [max]	22 (130) [4700]	5.4 (10) [150]	27 (130) [2300]	3.3 (14) [350]	26 (49) [11000]	2.8 (10) [410]	82 (160) [2100]	-
	**Naphthalene** ng m^{-3}	24	22	6.1	28	10	13	120	370
	**Fluorene** ng m^{-3}	2.1	1.9	1.4	2.3	2.7	2.4	1.5	4.1
	**Phenanthrene** ng m^{-3}	5.3	3.0	3.6	4.5	4.8	4.5	3.3	7.3
	**Pyrene** ng m^{-3}	2.3	1.3	1.5	3.5	1.6	1.8	1.8	2.5
	**Benzo[a]pyrene** ng m^{-3}	1.1	0.16	0.24	0.71	0.27	0.42	0.22	0.77
	**3 PAH** ng m^{-3}	9.7	6.1	6.5	10	9.1	8.8	6.7	14
	**16 US EPA PAH** ng m^{-3}	51	33	18	48	28	31	140	400

[a]Not measured

b)

Participant ID		*Asphalt millers*							
		1	***2***	***3***	***4***	***5***	***6***	***7***	***8***
**Urine samples**	**Measurement day**	A		B		C		D	
	**Work task**	Milling machine operator	Operator of sweeper	Milling machine operator	Operator of sweeper	**Work task**	Milling machine operator	Operator of sweeper	Milling machine operator
	**1-HP before work** µg/g Crea*	0.03	0.03	1.6	0.02	0.04	0.06	0.03	-[a]
	**1-HP after work** µg/g Crea	0.06	0.03	0.19	0.02	0.05	0.05	0.05	-
	**Sum 3 PAH** before work** µg/g Crea	0.51	0.47	2.8	0.29	0.42	0.54	2.7	-
	**Sum 3 PAH after work** µg/g Crea	0.41	0.33	1.3	0.23	0.35	0.36	1.0	-

[a]Not measured

*Creatinine

**Sum of metabolites of the three PAHs pyrene, fluorene and phenanthrene

Table S4. Individual average values of a) the personal air and b) urine samples of the asphalt pavers number 9-17. Standard deviation and the maximum value are also shown for the direct-reading measurements of eBC, LDSA, PNC, particle size, and $PM_{0.3}$. It can also be seen which measurements were carried out on which participant.

a)

		Asphalt pavers								
	**Participant ID**	***9***	***10***	***11***	***12***	***13***	***14***	***15***	***16***	***17***
**Air samples**	**Measurement day**	D					E			
	**Work task**	Roller operator	Screedman, vehicle operator, sampling of asphalt	Screedman, bitumen vehicle operator	Team leader, car driver, supervision	Shuttle buggy operator	Screedman	Paving machine operator	Screedman, wheel loader operator	Roller operator
	**Sampling time** min	432	513	448	446	481	733	757	752	729
	**eBC** µg m^{-3} (STD) [max]	-[a]	6.2	1.1	9.9	-	-	0.62	0.50	0.11
	**EC** µg m^{-3}	0.18	4.5	0.82	7.4	2.6	0.24	0.93	1.1	0.13
	**OC** µg m^{-3}	2.6	29	7.9	21	23	73	137	85	7.8
	**NO_2** µg m^{-3}	34	42	MD[b]	48	20	17	30	25	99
	**LDSA** µg m^{-3} (STD) [max]	-	82 (230) [4800]	19 (93) [2400]	-	-	-	150 (270) [2700]	100 (260) [3300]	35 (65) [1600]
	**PNC** cm^{-3} (STD) [max]	-	28000 (110000) [3100000]	8200 (47000) [1200000]	-	-	-	19000 (33000) [940000]	20000 (48000) [1600000]	11000 (15000) [340000]
	**Particle size** nm (STD) [max]	-	10 (61) [300]	70 (55) [300]	-		-	91 (83) [300]	60 (50) [300]	54 (33) [300]
	**$PM_{0.3}$** µg m^{-3} (STD) [max]	-	120 (540) [11000]	5.0 (25) [700]	-	-	-	420 (1100) [1300]	140 (530) [15000]	18 (100) [4100]
	**Naphthalene** ng m^{-3}	64	250	49	190	330	1100	4400	1700	260
	**Fluorene** ng m^{-3}	6.7	36	6.5	19	34	360	720	490	27
	**Phenanthrene** ng m^{-3}	4.8	43	8.2	24	45	360	700	490	25
	**Pyrene** ng m^{-3}	0.36	6.0	1.6	6.2	11	23	58	29	3.7
	**Benzo[a]pyrene** ng m^{-3}	<0.010	0.15	0.17	0.67	0.22	1.6	0.71	1.9	0.048
	**3 PAH** ng m^{-3}	12	85	16	49	90	740	1500	1000	56
	**16 US EPA PAH** ng m^{-3}	84	400	78	280	500	2100	6600	3100	340

[a]Not measured

[b]Missing data

b)

Participant ID		*Asphalt pavers*								
		9	***10***	***11***	***12***	***13***	***14***	***15***	***16***	***17***
**Urine samples**	**Measurement day**	D					E			
	**Work task**	Roller operator	Screedman, vehicle operator, sampling of asphalt	Screedman, bitumen vehicle operator	Team leader, car driver, supervision	Shuttle buggy operator	Screedman	Paving machine operator	Screedman, wheel loader operator	Roller operator
	**1-HP before work** µg/g Crea*	0.01	0.09	0.05	0.08	0.12	0.09	0.24	0.08	0.04
	**1-HP after work** µg/g Crea	0.03	0.12	0.04	0.1	0.23	0.16	0.23	0.20	0.04
	**Sum 3 OH-PAH** before work** µg/g Crea	0.39	0.81	0.34	0.65	0.82	1.2	1.3	0.69	0.32
	**Sum 3 OH-PAH after work** µg/g Crea	0.52	1.1	0.39	0.75	2.3	1.7	3.9	2.8	0.49

*Creatinine

**Sum of metabolites of the three PAHs pyrene, fluorene and phenanthrene

Table S5. Individual average values of a) the personal air and b) urine samples of the asphalt pavers number 18-26. Standard deviation and the maximum value are also shown for the direct-reading measurements of eBC, LDSA, PNC, particle size, and $PM_{0.3}$. It can also be seen which measurements were carried out on which participant.

a)

	**Participant ID**	***Asphalt pavers continue***								
		18	***19***	***20***	***21***	***22***	***23***	***24***	***25***	***26***
**Air samples**	**Measurement day**	F			G			H		
	**Work task**	Team leader, screedman	Roller operator	Paving machine operator	Asphalt spreader operator[a]	Asphalt paver, manually rake asphalt	Roller and ground vibrator operator, rake asphalt	Paving machine operator	Roller operator	Screedman
	**Sampling time** min	498	483	488	552	544	525	532	549	515
	**eBC** µg m^{-3} (STD) [max]	-[b]	0.46	0.27	0.47	1.5	2.2	1.3	1.5	2.5
	**EC** µg m^{-3}	0.40	0.59	0.19	0.34	0.65	2.3	0.52	2.0	1.1
	**OC** µg m^{-3}	25	14	16	19	19	11	29	22	28
	**NO_2** µg m^{-3}	17	21	19	27	20	34	37	55	41
	**LDSA** µg m^{-3} (STD) [max]	-	24 (86) [2000]	27 (52) [1400]	130 (700) [14000]	140 (550) [11000]	94 (230) [3500]	120 (340) [12000]	350 (830) [11000]	150 (470) [8800]
	**PNC** cm^{-3} (STD) [max]	-	25000 (130000) [3400000]	12000 (30000) [730000]	27000 (130000) [2700000]	42000 (220000) [8200000]	70000 (190000) [3200000]	38000 (240000) [17000000]	320000 (820000) [11000000]	71000 (350000) [5800000]
	**Particle size** nm (STD) [max]	-	10 (11) [150]	48 (25) [210]	52 (26) [300]	50 (25) [300]	36 (16) [260]	86 (81) [300]	33 (24) [300]	80 (70) [300]
	**$PM_{0.3}$** µg m^{-3} (STD) [max]	-	1.2 (4.0) [210]	7.7 (21) [380]	110 (1000) [78000]	82 (470) [1300]	94 (230) [3500]	200 (720) [20000]	23 (83) [2400]	150 (510) [7400]
	**Naphthalene** ng m^{-3}	110	31	76	50	47	24	170	170	120
	**Fluorene** ng m^{-3}	28	8.7	16	11	13	5.7	40	19	32
	**Phenanthrene** ng m^{-3}	40	14	25	19	23	10	72	35	66
	**Pyrene** ng m^{-3}	7.1	1.7	4.4	3.0	5.2	1.8	11	7.1	9.9
	**Benzo[a]pyrene** ng m^{-3}	0.086	0.011	0.038	0.24	0.035	0.018	0.085	0.15	0.14
	**3 PAH** ng m^{-3}	76	25	46	33	41	17	120	62	110
	**16 US EPA PAH** ng m^{-3}	220	66	150	110	110	53	360	270	280

[a]Also truck driver

[b]Not measured

b)

Participant ID		*Asphalt pavers continue* 18	*19*	*20*	*21*	*22*	*23*	*24*	*25*	*26*
Urine samples	**Measurement day**	F			G			H		
	**Work task**	Team leader, screedman	Roller operator	Paving machine operator	Asphalt spreader operator[a]	Asphalt paver, manually rake asphalt	Roller and ground vibrator operator, rake asphalt	Paving machine operator	Roller operator	Screedman
	**1-HP before** µg/g Crea*	0.15	0.03	0.19	0.05	0.02	0.02	0.03	0.08	0.08
	**1-HP after** µg/g Crea	0.09	0.03	0.14	0.04	0.06	0.05	0.13	0.19	0.17
	**Sum 3 OH-PAH* before** µg/g Crea	1.1	0.29	2.2	0.40	0.53	0.46	0.55	0.91	0.62
	**Sum 3 OH-PAH after** µg/g Crea	1.1	0.38	2.0	0.69	1.4	0.61	0.80	1.6	0.95

[a]Also truck driver
*Creatinine
**Sum of metabolites of the three PAHs pyrene, fluorene and phenanthrene

Table S6. Estimates of organic mass (OM) from measured organic carbon (OC). $PM_{0.3}$ measured with Partector 2 is also presented.

**Occupational group**	**Measured organic carbon (OC) GM* [min-max] (µg m^{-3})**	**Estimated organic mass (OM) GM [min-max] (µg m^{-3})**	**$PM_{0.3}$ GM [min-max] (µg m^{-3})**
Asphalt millers	40 [13-82]	48 [16-98]	13 [2.8-82]
Asphalt pavers	21 [3.0-140]	25 [3.6-170]	38 [1.2-420]

*Geometric mean

Table S7. Results from the correlation analysis of the exposure markers measured in the personal breathing zone. Spearman's rank correlation coefficient (r_s) is presented, with P-value shown in parentheses.

**Exposure marker**	**EC**	**OC**	**eBC**	**PNC**	**LDSA**	**$PM_{0.3}$**	**NO_2**	**Sum 3 PAH[a]**	**16 US EPA PAH**
**EC**	-	0.16 (0.449)	0.64 (0.002)**	0.70 (<0.001)**	0.48 (0.034)*	0.04 (0.885)	0.32 (0.129)	0.25 (0.233)	0.28 (0.184)
**OC**		-	-0.09 (0.700)	0.31 (0.190)	0.46 (0.041)*	0.38 (0.103)	-0.02 (0.933)	0.19 (0.359	0.17 (0.408)
**eBC**			-	0.43 (0.057)	0.36 (0.118)	0.34 (0.144)	0.22 (0.354)	0.27 (0.243)	0.29 (0.202)
**PNC**				-	0.75 (<0.001)**	0.13 (0.586)	0.23 (0.334)	0.20 (0.409)	0.08 (0.724)
**LDSA**					-	0.60 (0.005)**	0.07 (0.770)	0.37 (0.112)	0.39 (0.092)
**$PM_{0.3}$**						-	-0.25 (0.294)	0.61 (0.005)**	0.65 (0.002)**
**NO_2**							-	-0.36 (0.81)	-0.26 (0.213)
**Sum 3 PAH[a]**								-	0.865 (<0.001)**
**16 US EPA PAH**									-

[a]Sum of pyrene, fluorene and phenanthrene

*The correlation is significant at the 0.05 level (2-tailed)

**The correlation is significant at the 0.01 level (2-tailed)
